# Pygopus2 ameliorates mesenteric adipocyte poor differentiation to alleviate Crohn's disease ‐like colitis via the Axin2/GSK3β pathway

**DOI:** 10.1111/cpr.13292

**Published:** 2022-06-16

**Authors:** Jing Li, Lugen Zuo, Zhijun Geng, Qingqing Li, Yang Cheng, Zi Yang, Ruohan Shi, Yueqing Zhou, Wenhu Nie, Yueyue Wang, Xiaofeng Zhang, Sitang Ge, Xue Song, Jianguo Hu

**Affiliations:** ^1^ Department of Clinical Laboratory First Affiliated Hospital of Bengbu Medical College Bengbu China; ^2^ Department of Gastrointestinal Surgery First Affiliated Hospital of Bengbu Medical College Bengbu China; ^3^ Department of Central Laboratory First Affiliated Hospital of Bengbu Medical College Bengbu China; ^4^ Department of Clinical Medicine Bengbu Medical College Bengbu China

## Abstract

**Objectives:**

Crohn's disease (CD) mesenteric adipose tissue (MAT) inflammation affects enteritis through the interaction between the mesentery and intestine, and we previously found that poorly differentiated mesenteric adipocytes were related to its inflammatory features. Pygopus2 (Pygo2) is a key negative regulator of adipocyte differentiation. We aimed to determine whether Pygo2 participates in CD mesenteric lesions and whether Pygo2 knockdown would be beneficial in a CD model (*Il‐10* ^*−/−*^ mice).

**Methods:**

Pygo2 expression in MAT from control and CD patients and *Il‐10* ^*−/−*^ mice was measured by immunohistochemistry. Lentiviral transfection was used to regulate *Pygo2* expression in *Il‐10* ^*−/−*^ mice, and the effects on mesenteric adipocyte differentiation, inflammation, and dysfunction during spontaneous colitis, as well as the possible mechanism, were investigated.

**Results:**

Pygo2 expression was increased in MAT from CD patients and *Il‐10* ^*−/−*^ mice, and its expression correlated with poor adipocyte differentiation and inflammation. *Pygo2* knockdown significantly ameliorated colitis in *Il‐10* ^*−/−*^ mice. Moreover, the downregulation of *Pygo2* gene expression could promote adipocyte differentiation and inhibit adipocyte inflammation in vivo and in vitro, and the effects were at least partly mediated by the Axis inhibition protein 2 (Axin2)/glycogen synthase kinase 3 beta (GSK3β) pathway.

**Conclusions:**

The increase in Pygo2 may be related to mesenteric adipocyte poor differentiation and inflammatory features of CD, and Pygo2 inhibition could alleviate CD‐like colitis by improving mesenteric lesions by regulating the Axin2/GSK3β pathway.

## Graphical abstract

The increase in Pygopus2 (Pygo2) may be related to mesenteric adipocyte poor differentiation and inflammatory features of CD, and Pygo2 inhibition could alleviate adipocyte differentiation and mesenteric lesions by regulating the Axin2/GSK3β pathway.

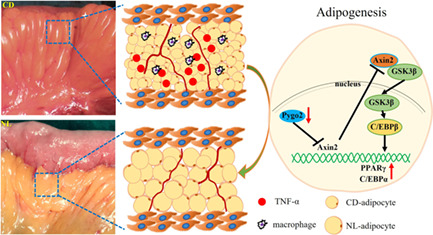

## 1 INTRODUCTION

Crohn's disease (CD) is characterized by chronic and relapsing intestinal inflammation, and hypertrophy of mesenteric adipose tissue (MAT) enveloping the lesions in the intestine.^1^, ^2^, ^3^ The crosstalk between MAT and the intestine affects the progression of CD.^4^ Studies have shown that the MAT in CD exhibits endocrine and metabolic dysfunction,^5^ as well as poor differentiation,^6^ and little is known about the underlying mechanism.

Adipose tissue is a multifunctional organ associated with lipid metabolism and endocrine functions that secretes a variety of soluble mediators, including adipokines and cytokines.^7^ The MAT in CD exhibits decreased expression of mature adipocyte markers (Perilipin, LPL, etc.), and lipid storage, fat synthesis, fat transport and other functions are impaired. Abnormally differentiated adipocytes recruit a large number of macrophages and form dysfunctional/necrotic adipocytes,^8^ which have the potential to induce and maintain acute or chronic adipose tissue inflammation, which may be the mechanism of CD‐associated mesenteric inflammation. Our previous research confirmed that mesentery functions can be improved by regulating the level of adipokines (Metrnl) in CD, including reducing mesenteric hypertrophy, improving adipocyte intrinsic function and ameliorating MAT inflammation, further relieving CD‐like colitis.^9^ Therefore, improving the abnormal differentiation of mesenteric adipocytes is expected to reduce intestinal inflammation and improve disease progression by alleviating mesenteric disease.

The differentiation of adipocytes is regulated by various molecules. The canonical Wnt/β‐Catenin signalling pathway is responsible for the activation of committed preadipocytes from mesenchymal stem cells but has an inhibitory effect on the differentiation of mature adipocytes. Pygopus2 (Pygo2) is a coactivator of the Wnt/β‐Catenin signalling pathway. The dynamic expression of Pygo2 is important for maintaining the development of various organs and manipulates the differentiation of multiple cell types.^10^, ^11^ Pygo2 was recently reported to mediate β‐Catenin activity in a gene‐ or tissue‐dependent manner in different physiological processes.^12^, ^13^ A meaningful study showed that Pygo2 expression decreased gradually during 3 T3‐L1 preadipocyte differentiation, and *pygo2* ^*−/−*^ mice exhibited spontaneous adipogenesis by inhibiting the expression of peroxisome proliferator‐activated receptor γ (PPARγ).^14^ However, the expression and contribution of Pygo2 in the pathogenesis of CD has not been elucidated. The effect of Pygo2 on the differentiation and maturation of adipocytes appears to reveal the possible mechanisms for CD‐associated mesenteric adipocyte differentiation and maturation disfunction. Thus, it is conceivable that Pygo2 interference can improve mesenteric adipocyte differentiation, and is to be considered as a treatment for CD mesenteric lesions.

In the present study, we aimed to measure the level of Pygo2 in CD MAT and to investigate whether Pygo2 influences the disease course of experimental colitis. We found that Pygo2 expression was significantly increased in the MAT of CD patients and *Il‐10* ^*−/−*^ mice compared with the MAT of controls. The absence of Pygo2 attenuated the development of MAT lesions in *Il‐10* ^*−/−*^ mice by promoting the differentiation of adipocytes, leading to the amelioration of colitis partly through the Axin2/GSK3β pathway. These results suggest a role for Pygo2 in manipulating mesenteric adipocyte differentiation and reducing inflammation in CD MAT and enrich the understanding of the crosstalk between MAT and CD pathogenesis.

## 2 MATERIALS AND METHODS

### 2.1 Patient specimens

MAT was collected from colon cancer patients (control) and patients with CD who underwent surgical resection (both n = 20). The study was approved by the Ethics Committee of the First Affiliated Hospital of Bengbu Medical College, and all patients provided informed consent during their preoperative visits. The enrolled CD patients had Montreal classifications of A2, L3 and B2 and underwent an initial ileocecal resection for stenosis [11 males and 9 females; mean age 31.3 (3.5) years; mean BMI 18.1 (1.1) kg/m^2^]. The control group included colon cancer patients [10 males and 10 females; mean age 63.2 (5.2) years; mean BMI 18.4 (0.7) kg/m^2^].

### 2.2 Animals

Wild‐type mice (WT) and *Il‐10* ^*−/−*^ mice on a C57BL/6J background were originally purchased from Model Animal Research Center of Nanjing University (Jiangsu Province, China) and raised or bred in a specific pathogen‐free (SPF) facility at Bengbu Medical College. The mice were housed 4–5 per cage and given access to food and water ad libitum. Fifteen‐week‐old *Il‐10* ^*−/−*^ mice consistently developed colitis when housed in an SPF environment as reported.^15^ The mice were sacrificed at the end of the experiment by cervical dislocation under isoflurane‐induced anaesthesia.

### 2.3 Pygo2 intervention and colitis symptom assessment

*Il‐10* ^*−/−*^ mice were infected with lentivirus by intravenous (IV) injection to overexpress *Pygo2* (*Pygo2* OE) or shRNA to knock down *Pygo2* (*Pygo2* KD). The sequences for *Pygo2*‐shRNA were 5′‐CCTGCGCCCCCCACTTTAG‐3′. *Il‐10* ^*−/−*^ mice were used as the model group, and WT mice were used as the control group (8 mice/group). At eight weeks of age (1 × 10^9^ viral particles/mouse/week, Shanghai GenePharma Co., Ltd), the model group received only PBS at the same time for 8 weeks. Changes in body weight were recorded weekly, and the inflammatory bowel disease activity index (DAI) was scored weekly as reported.^16^

### 2.4 In vivo imaging of intestinal inflammation

The luminescent probe L‐012 was used to evaluates the degree and distribution of colitis in mice by reacting with reactive oxygen species (ROS) generated during inflammation. As reported,^17^, ^18^ the animals were anaesthetised in an anaesthesia chamber with 2.0% isoflurane gas. After anaesthesia, the mice were intraperitoneally injected with 20 mmol L‐012 solution (100 μl/mouse, Wako Chemicals, Neuss, Germany). The mice were photographed 1 min after L‐012 injection, and the autoexposure option was used to allow the IVIS Spectrum CT bioluminescence imaging system (Perkin Elmer, Rodgau‐Jügesheim, Germany). automatically regulate acquisition parameters. The luminescent signal intensity indicates the degree of inflammation and the pseudo colours represent photons/s cm^2^ sr.

### 2.5 Histological examination

Haematoxylin–eosin (HE) staining of colon tissues and MAT from humans and mice was carried out as described previously.^19^ Briefly, mouse colon tissues were dissected into the proper size and embedded in paraffin after being fixed in formaldehyde solution for at least 24 h. MAT was fixed in special fixative for adipose tissue for at least 72 h (Servicebio, China). Sections were deparaffinized with xylene and rehydrated using standard procedures. HE staining was performed in 5 μm paraffin sections. The immunostaining score (0 to 4), which assessed inflammatory cell infiltration and tissue damage, was independently evaluated by three pathologists, and the detailed evaluation standard was reported.^20^, ^21^

### 2.6 Immunohistochemical analysis

Immunohistochemistry was performed as previously reported.^8^ Briefly, the tissues were paraffin‐embedded, dewaxed, and rehydrated. The sections were treated with primary antibodies (Perilipin, Pygo2, TNF‐α and F4/80; Abcam) overnight at 4°C. Then, HRP‐conjugated goat anti‐rabbit IgG was added dropwise and incubated, DAB substrate was used to develop the colour, and haematoxylin was used to stain the nuclei.

### 2.7 ELISA

The proinflammatory mediator (IFN‐γ, TNF‐α, IL‐6 and IL‐17A) levels in MAT and colon tissue extracts were measured by using a corresponding ELISA kit (eBioscience) according to the manufacturer's instructions. The tissue was washed with saline and weighed; 100 mg of tissue was homogenized with 1 ml of PBS. The extracts were obtained by homogenization with an electrical tissue homogenizer and centrifugation at 3500*g* for 20 minutes.

### 2.8 RT–qPCR

Total mRNA was extracted from MAT with TRIzol reagent (Invitrogen, USA), and the RNA concentration and purity were measured by a NanoDrop one microvolume UV–Vis Spectrophotometer (Thermo Fisher, USA). Complementary DNA (cDNA) was synthesized by a PrimeScript RT reagent kit (TAKARA, China). cDNA was subjected to RT–qPCR using SYBR green master mix (TAKARA, China) and analysed by the relative quantitative comparative threshold cycle (ΔΔCt) method. mRNA expression was normalized to GAPDH. The primer sequences are shown in Table 1.

**TABLE 1:** Primer sequences [5′ to 3′]

Gene	Forward primer	Reverse primer
IFN‐γ	ACAGCAAGGCGAAAAAGGATG	TGGTGGACCACTCGGATGA
IL‐17A	GGCCCTCAGACTACCTCAAC	TCTCGACCCTGAAAGTGAAGG
TNF‐α	CAGGCGGTGCCTATGTCTC	CGATCACCCCGAAGTTCAGTAG
IL‐1β	GAAATGCCACCTTTTGACAGTG	TGGATGCTCTCATCAGGACAG
IL‐6	CTGCAAGAGACTTCCATCCAG	AGTGGTATAGACAGGTCTGTTGG
Nos2	GTTCTCAGCCCAACAATACAAGA	GTGGACGGGTCGATGTCAC
CD274	AGTATGGCAGCAACGTCACG	TCCTTTTCCCAGTACACCACTA
Arg1	AGACCACAGTCTGGCAGTTG	CCACCCAAATGACACATAGG
CD206	TGATTACGAGCAGTGGAAGC	GTTCACCGTAAGCCCAATTT
Pygo2	AGCGAAGAAAGTCCAATACTCAG	GTTAGAAGCGACCAGATGATCC
GAPDH	AGGTCGGTGTGAACGGATTTG	TGTAGACCATGTAGTTGAGGTCA

### 2.9 3 T3‐L1 preadipocyte culture and lentiviral infection

The murine 3 T3‐L1 preadipocyte cell lines were purchased from ATCC (Lot:CL‐173; USA) and was not used past passage 10. Undifferentiated 3 T3‐L1 cells were grown in DMEM supplemented with 10% newborn calf serum (Gibco, USA), penicillin (100 U/mL) and streptomycin (0.1 mg/mL) at 37°C with 5% CO_2_. The differentiation of 3 T3‐L1 preadipocytes to adipocytes was conducted as previously reported.^22^ In brief, the cells were allowed to grow for 2 d after reaching confluence and were differentiated by the addition of hormonal cocktails, including 5 μM dexamethasone (Sigma–Aldrich, USA), 0.5 mM 3‐isobutyl‐1‐methylxanthine (Sigma–Aldrich, USA), and 5 μg/mL bovine insulin (Sigma–Aldrich, USA), for 3 d. Then, the medium, which contained only 10% fetal bovine serum and bovine insulin, was changed every 2 d. For the lipopolysaccharide (LPS)‐stimulated 3 T3‐L1 cell experiment, during cell differentiation into adipocytes, additional LPS (10 μg/mL) was added to the medium as previously reported.^23^

For *Pygo2* gene intervention, 3 T3‐L1 cells (8–10 d after differentiation) were infected with lentiviruses to mediate *Pygo2* knockdown and *Pygo2* overexpression (Shanghai GenePharma Co., Ltd.). After incubation for 4 hours, the medium was changed. The knockdown and overexpression efficiency were verified by Western blotting.

### 2.10 Oil red O staining

After the indicated intervention for 14 d, the differentiated 3 T3‐L1 cells were fixed with formaldehyde as previously described and stained with filtered Oil red O (Sigma–Aldrich, USA). The oil droplets were photographed with a microscope (Olympus).

### 2.11 Western blotting

The proteins in MAT and 3 T3‐L1 cells were analysed by Western blotting. MAT protein was extracted using an Adipose Protein Extraction Kit (MinuteTM). A subcellular protein extraction kit (Millipore) was used to extract nuclear proteins. Both MAT and 3 T3‐L1 protein lysates underwent SDS‐PAGE and were transferred to polyvinylidene difluoride (PVDF) membranes. Then, the membranes were blocked with 5% nonfat dry milk and probed with primary antibodies against ATGL, LPL, Pygo2, Perilipin, Axin2, GSK3β, PPARγ, C/EBPα, C‐myc, Cyclin D1, laminB, and β‐actin. The membrane was then blotted with the anti‐rabbit/mouse immunoglobulin G secondary antibodies (dilution 1:5000; Abcam) in blocking buffer for 1 hour and visualized by autoradiography.

### 2.12 Statistical analysis

All experiments were performed at least three times independently, and representative data are displayed. Data analyses were performed using GraphPad Prism version 9.3.1. Continuous normally distributed data are presented as the mean ± standard deviation (SD) and were analysed by unpaired two‐tailed Student's *t* tests. Correlation analysis was performed using Pearson correlation analysis. *p* < 0.05 was considered statistically significant.

## 3 RESULTS

### 3.1 The increase in Pygo2 in the MAT of CD patients correlated with mesenteric adipocyte poor differentiation and inflammation

**FIGURE 1:**
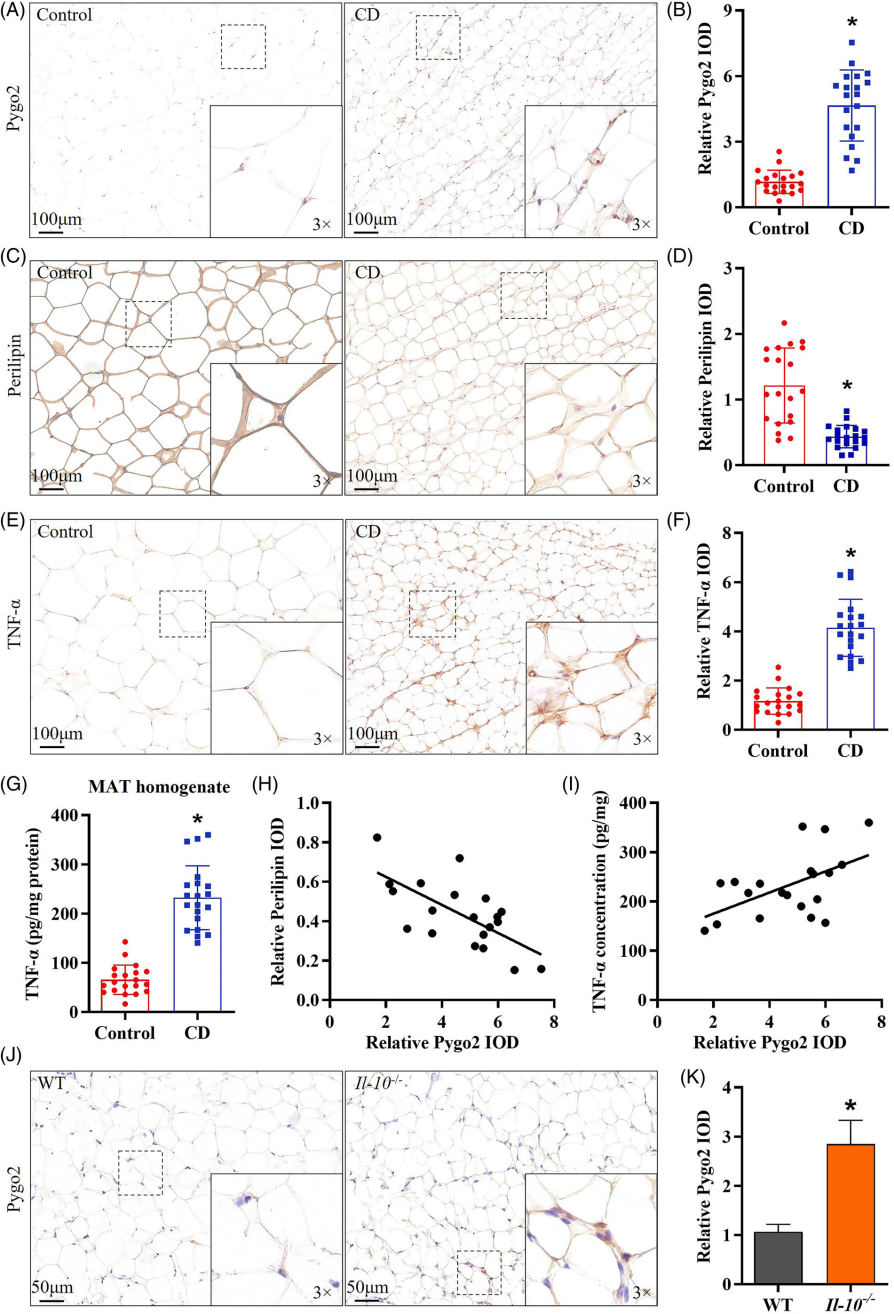
Increased Pygo2 in the MAT of CD patients and *Il‐10* ^*−/−*^ mice. Representative image and quantitative analysis of Pygo2 (A‐B), Perilipin (C‐D), and TNF‐α (E‐F) expression in the MAT of control patients and CD patients, as shown by immunohistochemical staining (both n = 20). ELISA analysis of TNF‐α in the MAT homogenate of control patients and CD patients (G). Correlation analysis of the relative protein expression of Pygo2, Perilipin (H) and TNF‐α (I) in CD patients. Immunohistochemical staining (J) and quantitative analysis (K) of Pygo2 in the MAT of *Il‐10* ^*−/−*^ mice and WT mice. The dot plots are representative of 8 independent animals. Pygo2, Pygopus2; MAT, mesenteric adipose tissue; CD, Crohn's disease; WT, wild‐type; IOD, integrated optical density; *Il‐10* ^*−/−*^, *Il‐10* deficient. The data are presented as the mean ± SD. *p < 0.05

CD patient MAT was characterized by differentiation and functional abnormalities.^8^ Pygo2 is a negative regulator of adipocyte differentiation.^14^ We found that the expression of Pygo2 in the MAT of CD patients was significantly increased compared with that in the controls (Figure 1A‐B). Moreover, CD MAT showed decreased expression of Perilipin (Figure 1C‐D) and increased levels of TNF‐α (Figure 1E‐G). Further analysis showed that the expression of Pygo2 in CD exhibited the opposite pattern with adipocyte differentiation (Perilipin; Figure 1H) and positively correlated with mesenteric inflammation (TNF‐α; Figure 1I). The increased Pygo2 may explain the poor differentiation and dysfunction of CD mesenteric adipocytes. In addition, we also found that the expression of Pygo2 in the MAT of the CD mouse model (*Il‐10* ^*−/−*^ mice) was also significantly increased compared with that in WT mice (Figure 1J‐K). Next, we used *Il‐10* ^*−/−*^ mice to examine the role and potential mechanism of Pygo2 in CD.

### 3.2 Pygo2 knockdown ameliorated spontaneous colitis in Il‐10^−/−^ mice

**FIGURE 2:**
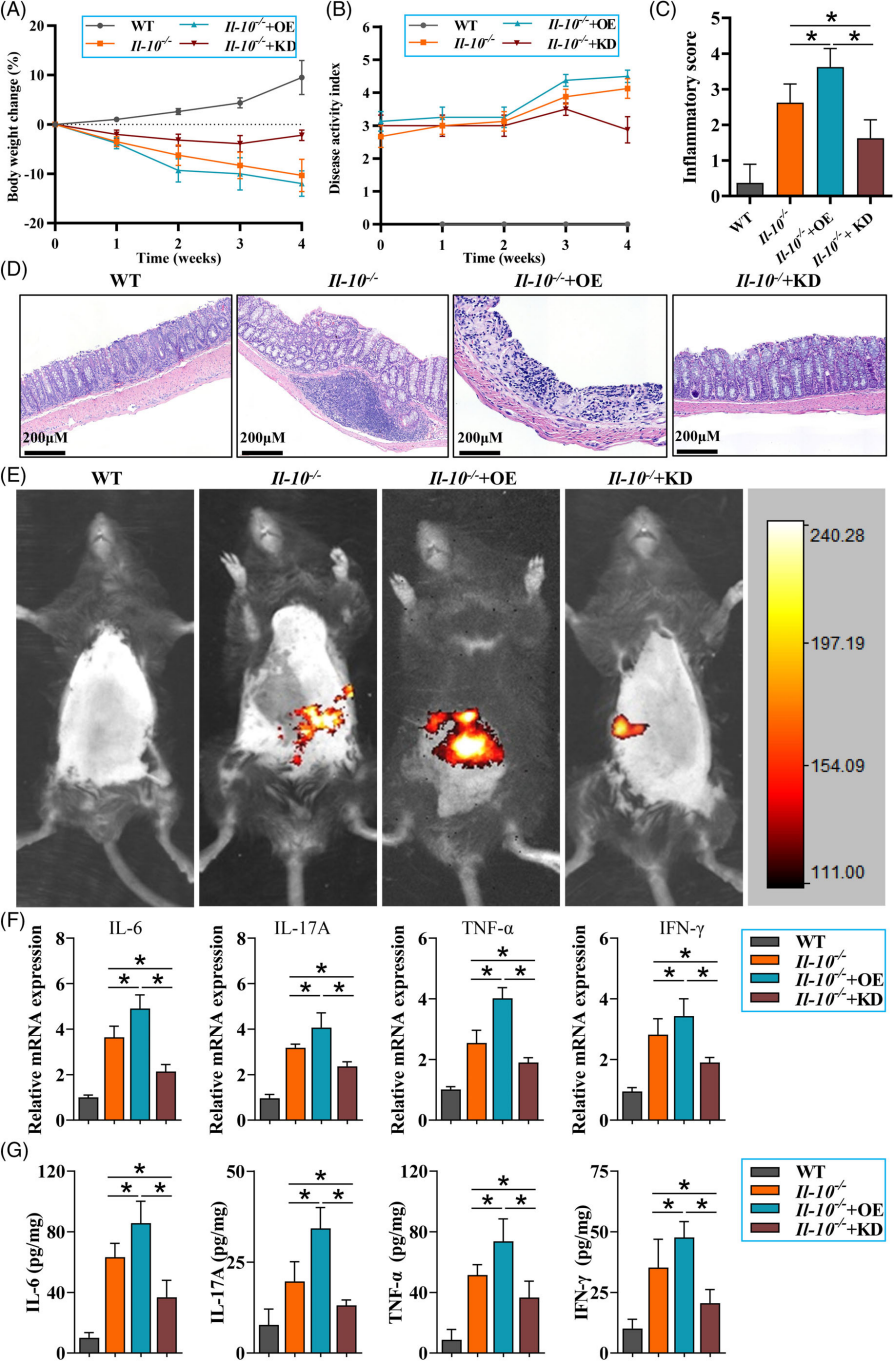
Pygo2 knockdown ameliorated clinical signs of colitis in *Il‐10* ^−/−^ mice. *Il‐10* ^−/−^ mice were infected with lentivirus by intravenous (IV) injection to induce Pygo2 overexpression (OE) or shRNA to induce Pygo2 knockdown (KD); *Il‐10* ^*−/−*^ mice were the model group, and WT mice were the control group (8 mice/group). The mice were treated at eight weeks of age for 4 weeks (1 × 10^9^ viral particles/mouse/week). The percentage change in body weight in WT, *Il‐10* ^*−/−*^ *and Il‐10* ^*−/−*^ Pygo2 OE or Pygo2 KD mice (A). The disease activity index (DAI) values in the four groups were observed every week (B). Representative pathological images stained with H&E and inflammation scores of colon tissues (C‐D). In vivo imaging of intestinal inflammation and representative bioluminescent images are shown (E). The mRNA and protein levels of inflammatory mediators (IL‐6, IL‐17A, TNF‐α and IFN‐γ) were measured by RT–qPCR (F) and ELISA (G). WT, wild‐type; IL‐6, interleukin‐6; IL‐17A, interleukin‐17A; TNF‐α, tumour necrosis factor‐α; IFN‐γ, interferon‐γ; OE, Pygo2 overexpression; KD, Pygo2 knockdown. The data are expressed as the mean ± SD. *p < 0.05.

To examine the role of Pygo2 in CD‐like colitis, we used lentivirus to specifically knock down (KD) or overexpress (OE) *Pygo2* in *Il‐10* ^*−/−*^ mice and the intervention effect in MAT and intestinal mucosa was verified by RT‐qPCR (Figure S1). The results showed that *Pygo2* KD in *Il‐10* ^*−/−*^ mice resulted in significantly less weight loss (Figure 2A) and lower DAI scores (Figure 2B) than those of *Il‐10* ^*−/−*^ mice at 4 weeks after intervention. On the other hand, *Pygo2*‐overexpressing *Il‐10* ^*−/−*^ mice showed more severe clinical signs than those in the other groups. The intestinal histopathology and inflammatory scores were also significantly decreased in *Pygo2* KD *Il‐10* ^*−/−*^ mice compared with the others (Figure 2C‐D). In addition, live imaging of mice and inflammatory mediator (IL‐6, IL‐17A, TNF‐α, IFN‐γ) levels in the intestinal mucosa were significantly reduced in *Pygo2* KD *Il‐10* ^*−/−*^ mice compared with *Il‐10* ^*−/−*^ mice and *Pygo2* OE *Il‐10* ^*−/−*^ mice (Figure 2E‐G). Our data indicated that *Pygo2* knockdown could alleviate CD‐like colitis in *Il‐10* ^*−/−*^ mice.

### 3.3 Pygo2 knockdown attenuated mesenteric inflammation in Il‐10^−/−^ mice

**FIGURE 3:**
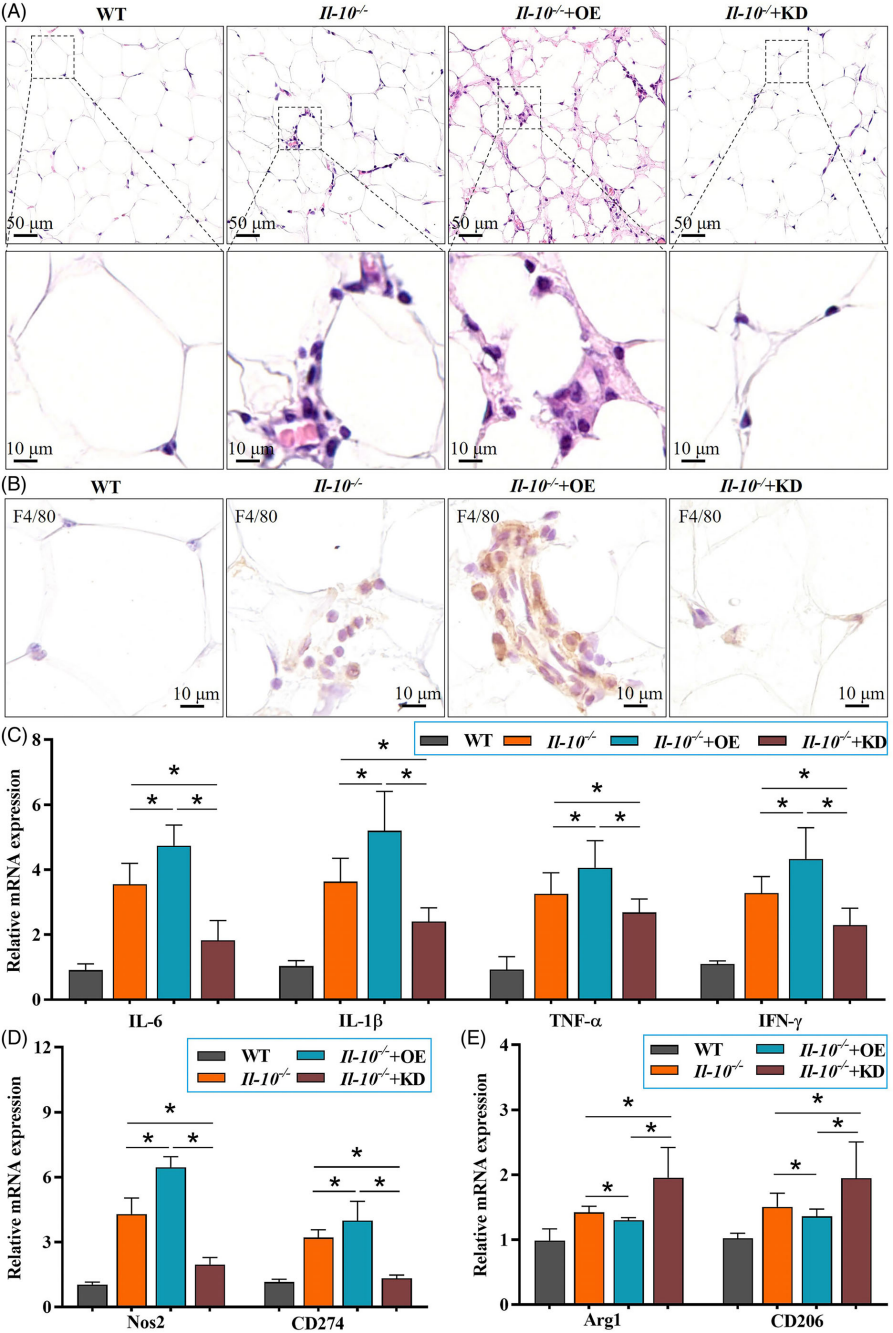
Pygo2 knockdown attenuated mesenteric inflammation in *Il‐10* ^*−/−*^ mice. *Il‐10*−/− mice were treated and divided into four groups as shown in Figure 2 (both n = 8). Representative pathological images of MAT in WT, *Il‐10* ^*−/−*^ and *Il‐10* ^*−/−*^ Pygo2 OE or Pygo2 KD mice (H&E staining, A). Immunohistochemical staining of F4/80‐positive macrophages in the MAT (B). The mRNA levels of inflammatory mediators (IL‐6, IL‐1β, TNF‐α and IFN‐γ) in MAT were measured by RT–qPCR (C). The mRNA levels of M1 macrophage markers (Nos2 and CD274, D) and M2 macrophage markers (Arg1 and CD206, E) in MAT were measured by RT–qPCR. WT, wild‐type; IL‐6, interleukin‐6; IL‐1β, interleukin‐1β; TNF‐α, tumour necrosis factor‐α; IFN‐γ, interferon‐γ; OE, overexpression; KD, knockdown. The data are expressed as the mean ± SD. *p < 0.05.

Then, we further analysed the effect of Pygo2 on mesenteric inflammation, which could interact with colitis during the progression of the disease. Compared with those of WT mice, pathological sections of MAT from *Il‐10* ^*−/−*^ mice showed severe inflammation (Figure 3A) and increased F4/80^+^ macrophage infiltration (Figure 3B), which was consistent with previous studies.^9^ *Pygo2* KD *Il‐10* ^*−/−*^ mice exhibited mild MAT inflammation and less macrophage infiltration than *Il‐10* ^*−/−*^ mice and *Pygo2* OE *Il‐10* ^*−/−*^ mice (Figure 3A‐B). The levels of proinflammatory mediators (IL‐6, IL‐1β, TNF‐α, IFN‐γ) were consistent with previous results (Figure 3C). Macrophages in adipose tissue tend to have an anti‐inflammatory phenotype, which is indicated by decreased expression of M1 macrophage markers (Nos2 and CD274) and increased expression of M2 macrophage markers (Arg1 and Mrc‐1) in *Pygo2* KD mice compared with *Il‐10* ^*−/−*^ and *Pygo2* OE *Il‐10* ^*−/−*^mice (Figure 3D‐E). These results demonstrated that the absence of Pygo2 could ameliorate mesenteric inflammation.

### 3.4 Pygo2 knockdown improved mesenteric adipocyte differentiation in Il‐10^−/−^ mice

**FIGURE 4:**
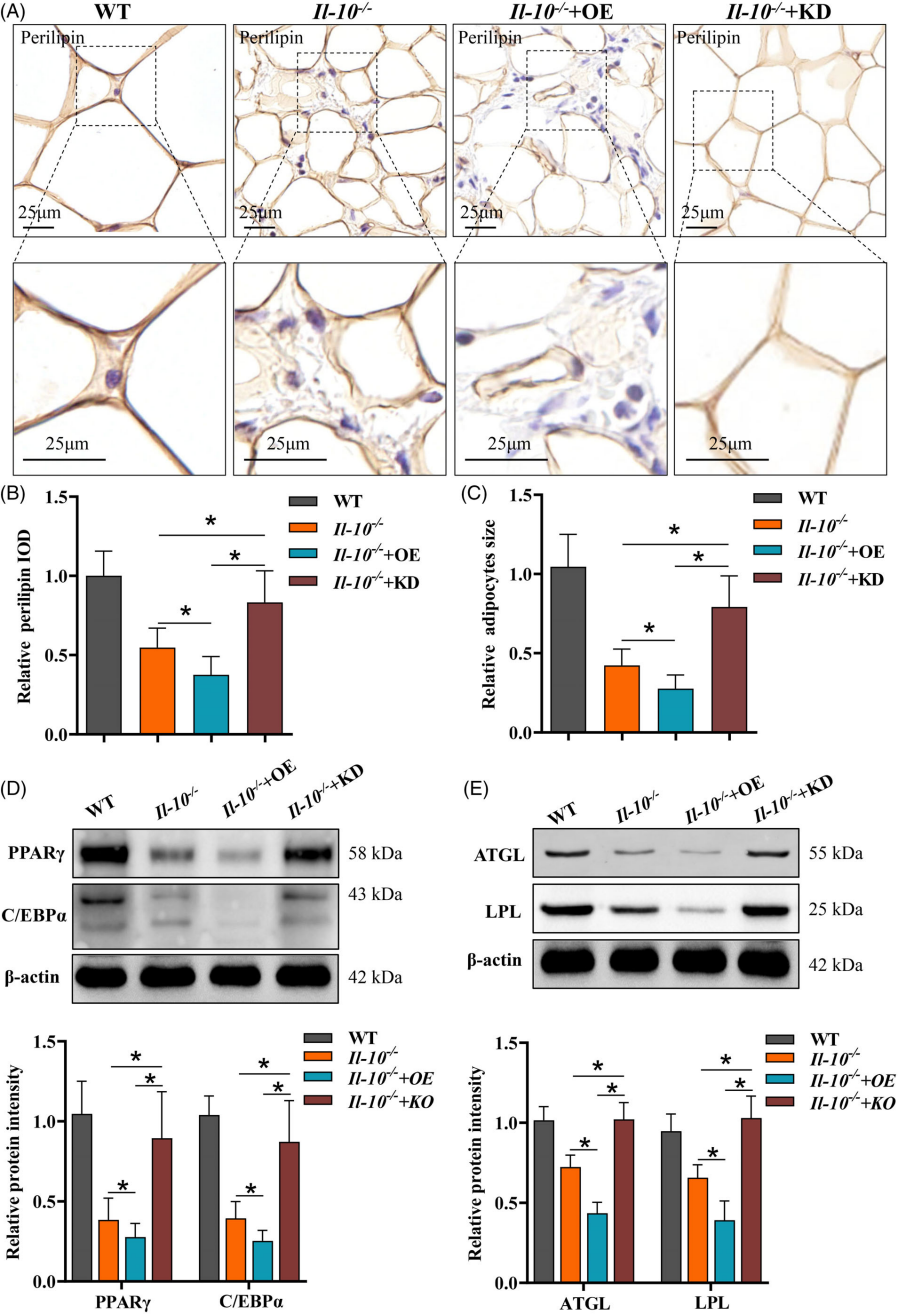
Pygo2 knockdown ameliorated mesenteric adipocyte poor differentiation in *Il‐10* ^*−/−*^ mice. *Il‐10* ^−/−^ mice were treated and divided into four groups as shown in Figure 2 (both *n* = 8). Immunohistochemical staining of the adipocyte maturation marker perilipin in MAT (A). Quantitative analysis of perilipin expression in MAT (B). Relative adipocyte size (C). Western blot and relative protein intensities of PPARγ and C/EBPα (D). Western blot and relative protein intensities of ATGL and LPL (E). The relative protein intensity was evaluated by ImageJ software, and the IOD values of the target protein were normalized to β‐actin. PPARγ, peroxisome proliferator‐activated receptor γ; C/EBPα, CCAAT/enhancer binding protein (C/EBP) alpha; ATGL, adipose triglyceride lipase; LPL, lipoprotein lipase; IOD, integrated optical density; WT, wild‐type; OE, overexpression; KD, knockdown. The data are expressed as the mean ± SD. *p < 0.05

Previous studies have suggested that adipocyte dysfunction and poor differentiation could aggravate CD colitis.^24^ Considering the inhibitory effect of Pygo2 on adipocyte differentiation, we examined whether the absence of Pygo2 attenuated inflammation in the intestine and MAT by promoting the differentiation of adipocytes in CD. We found that *Pygo2* KD *Il‐10* ^*−/−*^ mice showed increased expression of perilipin, a marker of mature adipocytes (Figure 4A,B). In addition, the size of adipocytes was also significantly recovered in *Pygo2* KD mice compared with *Il‐10* ^*−/−*^ mice (Figure 4C). PPARγ is a member of the nuclear hormone receptor superfamily that was originally shown to play a critical role in adipocyte differentiation and lipid metabolism^25^ and has recently been implicated in inhibiting inflammation in MAT.^9^ C/EBPα can activate its own transcription and the expression of PPARγ. The presence of PPARγ and C/EBPα can maintain the expression of mature adipocyte genes, such as ATGL and LPL.^26^ Our data showed that *Pygo2* knockdown augmented the expression of PPAR‐γ and C/EBPα in *Il‐10* ^*−/−*^ mice (Figure 4D), which was concomitant with increased expression of ATGL and LPL (Figure 4E). These data indicate that *Pygo2* knockdown enhances the differentiation of adipocytes in CD mice.

### 3.5 Pygo2 inhibited adipocyte differentiation in vitro through the Axin2/GSK3β pathway

**FIGURE 5:**
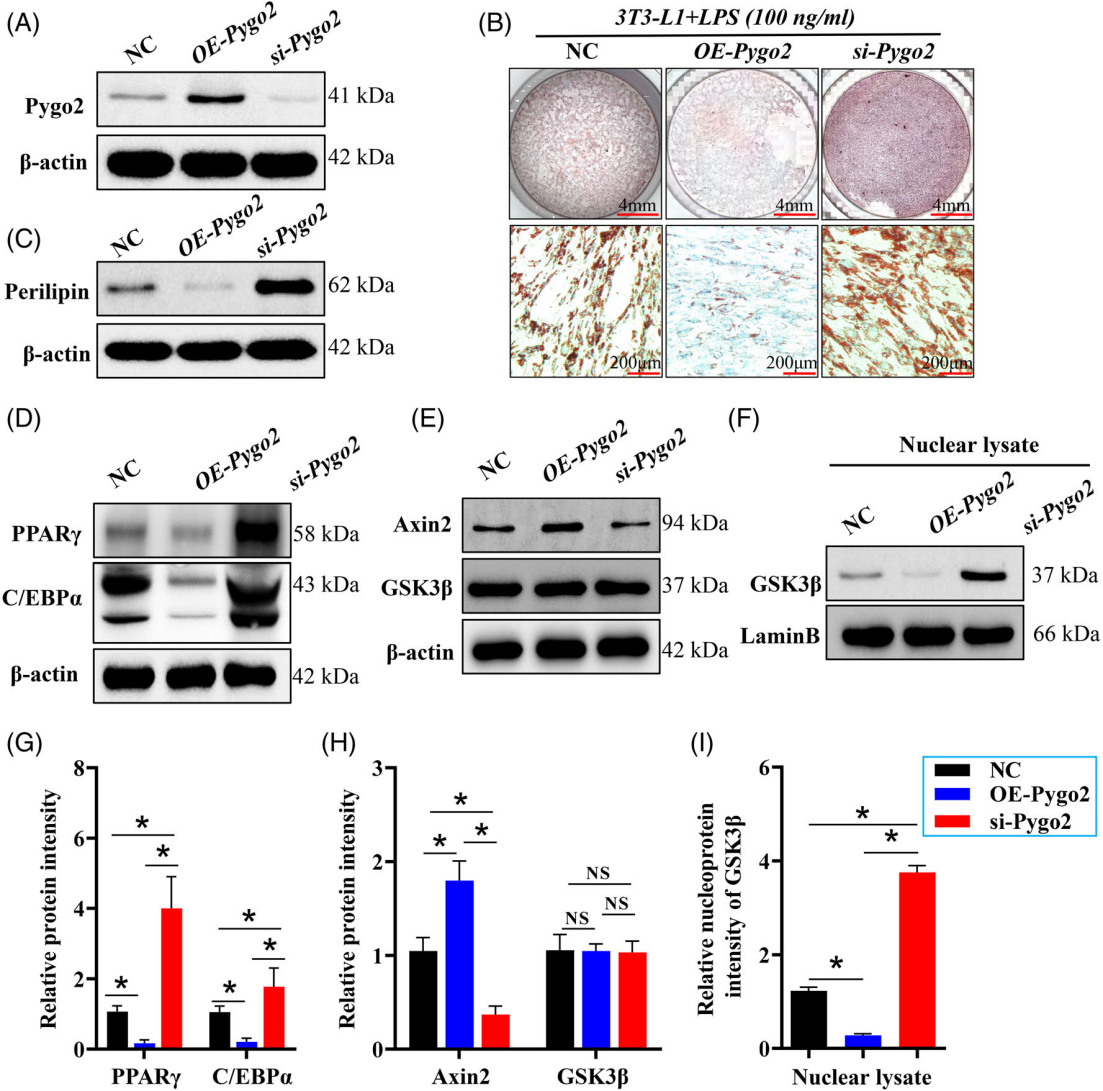
Pygo2 regulates adipocyte differentiation and interacts with the Axin2/GSK3β pathway in the CD context. The 3 T3‐L1 cells (8–10 d after differentiation) were infected with lentiviruses to mediate si‐Pygo2 and Pygo2 overexpression (OE‐Pygo2) and stimulated with LPS (100 ng/mL). The efficiency of knockdown and overexpression was verified by Western blotting at 14 d after differentiation (A). The cells were fixed and stained with Oil red O, and the representative image was shown (B). Total and nuclear proteins were then extracted from the cells, and Western blot analysis of Perilipin was performed (C). Western blot analysis of PPARγ and C/EBPα (D), as well as Axin2 and GSK3β (E), in total MAT protein. Western blot analysis of GSK3β in the nucleus in MAT (F). Relative intensities of the proteins (G‐I). PPARγ, peroxisome proliferator‐activated receptor γ; C/EBPα, CCAAT/enhancer binding protein (C/EBP) alpha; Axin2, Axis inhibition protein 2; GSK3β, glycogen synthase kinase 3 beta. The data are expressed as the mean ± SD. *p < 0.05.

We further explored the mechanism by which Pygo2 regulates adipocyte differentiation in the CD niche. We used LPS to stimulate 3 T3‐L1 cells and construct an inflammation model and examined the mechanism by which Pygo2 regulates adipocyte differentiation in the CD inflammatory environment. A lentivirus was used to specifically knock down (si‐*Pygo2*) or overexpress *Pygo2* (OE‐*Pygo2*) levels in 3 T3‐L1 cells. The NC group was infected with a control virus, and the intervention effect was verified by Western blotting (Figure 5A). 3 T3‐L1 cells with si‐*Pygo2*, OE‐ *Pygo2* or NC were then induced to differentiate into mature adipocytes in the presence of the full differentiation cocktail (MDI) and LPS (100 ng/mL) for 14 days. As shown in Figure 5B, 3T3‐L1 cells infected with si‐*Pygo2* effectively accumulated lipid droplets compared with NC and OE‐*Pygo2* cells and exhibited increased expression of perilipin in the presence of LPS (Figure 5C). This observation was consistent with the increased protein expression of PPARγ and C/EBPα in si‐*Pygo2* 3 T3‐L1 cells compared with the other groups (Figure 5D,G). Furthermore, OE‐*Pygo2* strongly inhibited the accumulation of lipid droplets and adipocyte differentiation.

To answer the effect of pygo2 on regulating adipocyte differentiation in CD model mice whether related to its role as a coactivator of the Wnt signalling pathway. We regulated the expression of Pygo2 in LPS‐stimulated 3 T3‐L1 cell and found that overexpression of Pygo2 could significantly activate the Wnt signalling pathway, manifested as increased expression of Axin2 (Figure 5E,H) and the target molecule (c‐myc and cyclin D1, Figure S2), when interfering with pygo2, the activation of Wnt signalling pathway was significantly inhibitor (Figure 5E and Figure S2). In addition, under the condition of Wnt signalling pathway activation, the increased Axin2 binding to GSK3β, and restricts GSK3β nuclear translocation. This may results in a significant inhibition of the ability of GSK3β to promote the expression of C/EBPα and PPARγ (Figure 5D,G) and as reported.^14^, ^27^ However, we verified in Figure 5F, si‐*Pygo2* facilitated GSK3β nuclear translocation (Figure 5F,I). The enrichment of GSK‐3β in the nucleus facilitates adipocyte differentiation in CD. These results indicated that pygo2 manipulates the process of adipocyte differentiation partly by regulating the Axin2/GSK3β signalling pathway.

### 3.6 Pygo2 inhibited mesenteric adipocyte differentiation in Il‐10^−/−^ mice through the Axin2/GSK3β pathway

**FIGURE 6:**
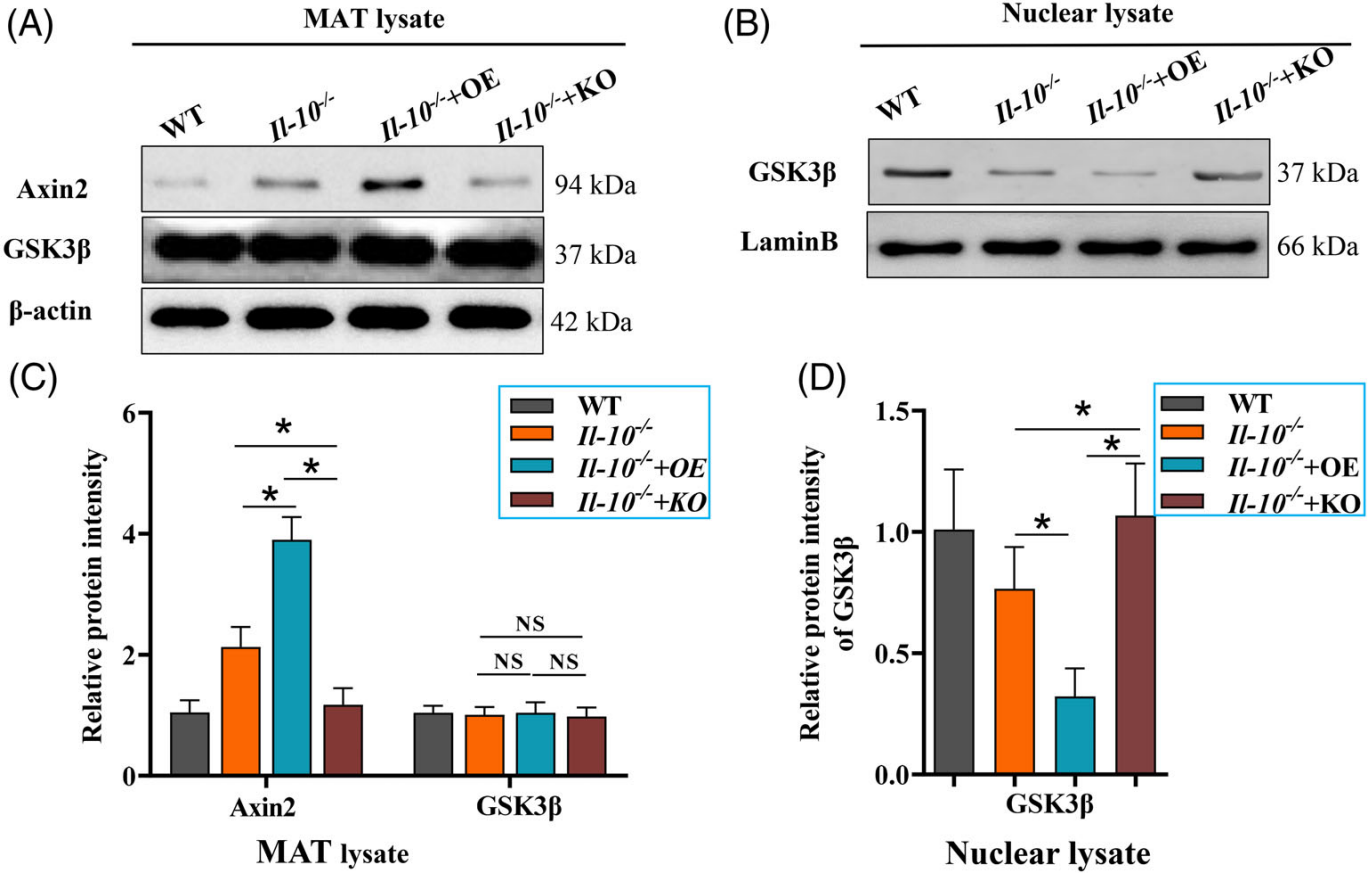
Pygo2 inhibits adipocyte differentiation in the MAT of *Il‐10* ^*−/−*^ mice through the Axin2/GSK3β pathway *Il‐10* ^−/−^ mice were infected with lentivirus by intravenous (IV) injection to induce Pygo2 overexpression (Pygo2 OE) or shRNA to induce Pygo2 knockdown (Pygo2 KD). *Il‐10* ^*−/−*^ mice were used as the model group, and WT mice were used as the control group (8 mice/group). The mice were treated at eight weeks of age for 8 weeks (1 × 10^9^ viral particles/mouse/week). The mice were sacrificed, the MAT was isolated, and total and nuclear proteins were extracted. Western blot analysis of Axin2 and GSK3β in total MAT proteins (A). Western blot analysis of GSK3β in the nucleus in MAT (B). Relative intensities of the proteins (C‐D). The data are expressed as the mean ± SD. *p < 0.05

Finally, we verified the mechanism by which Pygo2 regulates the differentiation of mesenteric adipocytes in CD mice. We examined the Axin2/GSK3β pathway in the MAT of WT, *Il‐10* ^*−/−*^ and *Il‐10* ^*−/−*^ mice with *Pygo2* KD or OE. We found that *Pygo2* KD *Il‐10* ^*−/−*^ mice showed decreased Axin2 and increased GSK3β nuclear expression compared to *Il‐10* ^*−/−*^ mice and *Pygo2* OE mice (Figure 6A‐D). These data indicated that Pygo2 inhibited adipocyte differentiation and maturation in the MAT of *Il‐10* ^*−/−*^ mice partly via the Axin2/GSK3β pathway.

## 4 DISCUSSION

In this study, we examined the role of Pygo2 in the regulation of adipocyte differentiation in CD in vitro and in vivo. We found increased expression of Pygo2 in the MAT of CD patients and *Il‐10* ^*−/−*^ mice, which was negatively correlated with adipocyte differentiation and positively correlated with mesenteric inflammation. The absence of Pygo2 attenuated mesenteric inflammation and adipocyte differentiation, which eventually ameliorated the clinical signs of colitis in *Il‐10* ^*−/−*^ mice. Further analysis showed that Pygo2 regulated adipocyte differentiation and interacted with the Axin2/GSK3β pathway in CD.

The most important finding in the study was that Pygo2 was increased in CD‐MAT and participated in adipocyte differentiation following colitis. A recent meaningful study found that Pygo2 can inhibit adipocyte differentiation in a high‐fat feeding‐induced mouse obesity model.^14^ In the visceral adipose tissue of obese patients, the number of adipocytes is basically unchanged, but the volume becomes larger. However, there are unique pathological changes in the CD mesenteric with increased number of adipocytes and small volume.^21^, ^28^ Given the function of Pygo2 in inhibiting adipocyte differentiation and lipid storage, we speculate that it may explain the unique pathological changes of CD mesenteric adipocytes. The poorly differentiated adipocytes may be the main culprit of mesenteric fat inflammation and the aggravation of CD colitis.^5^ This finding gives us a hint that if the poor differentiation of mesenteric adipocytes is improved, mesenteric lesions will be reduced. We found for the first time that the expression of Pygo2, an important transcription factor that regulates adipocyte differentiation,^14^ was increased in CD MAT, and the level was more than 4 times higher than that in the WT mice. It is interesting to note that the level of Pygo2 in CD showed the opposite pattern with adipocyte differentiation (Perilipin) and positively correlated with mesenteric inflammation (TNF‐α). We hypothesize that Pygo2 may be a critical molecule that leads to the poor differentiation of adipocytes in CD. We used lentivirus to specifically knock down and overexpress *Pygo2* in CD model mice and investigated its role. We found that *Pygo2* knockdown promoted the differentiation of adipocytes. Moreover, the expression of differentiation transcription factors (PPARγ and C/EBPα) and markers of mature adipocytes (ATGL and LPL) was increased. Interestingly, the volume of adipocytes returned to a nearly normal size. Further research showed that mesenteric inflammation was also significantly alleviated. Relatedly, the adipocytes *of Il‐10* ^*−/−*^ mice that overexpressed *Pygo2* showed dysfunction, poor differentiation, abnormal morphology and aggravated inflammation. In addition, we found that after Pygo2 intervention, the endocrine function and lipid metabolism of adipocytes were improved (data not shown). Our results indicate that high Pygo2 expression may be an important cause of poor adipocyte differentiation in CD. Inhibiting Pygo2 is expected to ameliorate mesenteric inflammation and can be a possible therapeutic strategy for CD.

In view of the crosstalk between MAT and the diseased intestine in patients with CD,^3^ mesenteric intervention can be an important means to treat CD colitis. Our results also confirmed that by regulating the expression of Pygo2, the symptoms of colitis in *Il‐10* ^*−/−*^ mice were significantly alleviated, as indicated by decreases in weight loss, DAI scores, and the infiltration of inflammatory cells in the intestine, and the alleviation of epithelial cell damage and mucosal edema. In addition, the recruitment and polarization of macrophages were also changed, the level of inflammation decreased, and the polarization of macrophages skewed toward the alternatively activated M2 phenotype. Our results confirmed that Pygo2 could reduce CD colitis by improving mesenteric adipocyte function and inflammation.

Pygo2 is an important member of the Wnt/β‐catenin pathway that can promote proliferation and inhibit differentiation.^29^ It was also observed in our study that overexpression of pygo2 in LPS stumilated‐3 T3‐L1 cells can promote the activation of Wnt signalling, and the expression of the downstream target genes (c‐myc, cyclin D1 and Axin2). While interfering with pygo2, the activation of the Wnt signalling was significantly inhibited, manifested as decreased expression of c‐myc, cyclin D1 and Axin2.The data indicates that Axin2 is a good Wnt target gene helpful to monitoring the activation status of Wnt signalling, of which Pygo2 is a component. Ours and previous studies have confirmed that Pygo2 can regulate the differentiation of adipocytes by regulating the expression of Axin2 and the nuclear transfer of GSK3β in a Wnt‐independent pathway. Our research showed that after inhibiting Pygo2 in *Il‐10* ^*−/−*^ mice, the inhibition of differentiation was reversed, GSK3β nuclear translocation occurred, and the expression of PPARγ and C/EBPα was increased. In addition to being an important transcription factor for adipocyte differentiation, PPARγ also has an anti‐inflammatory effect. In CD animal models, increased levels of PPARγ can directly exert anti‐inflammatory effects and thereby mitigate enteritis.^30^, ^31^ Therefore, *Pygo2* knockdown in *Il‐10* ^*−/−*^ mice can promote PPARγ expression and induce the differentiation of adipocytes, while also inhibiting mesenteric fat inflammation, thereby inhibiting colitis. Therefore, based on our findings, it is believed that Pygo2 may regulate the nuclear transfer of GSK3β through the Wnt/Axin2 pathway and the tranc downstream target genes, including the key molecules of adipocyte differentiation PPARγ and C/EBPα. Thus play a role in regulating adipocyte differentiation. But we cannot ignore the Wnt‐independent role of Pygo2, such as its activity in regulating the lens development^32^, ^33^ and tooth enamel formation.^32^ More studies have found that Pygo2 regulates mouse embryonic development mainly by binding and interacting with H3K4me2/3, but not Wnt‐dependent function.^34^

This study did not explore what factors in the CD environment increase the expression of Pygo2 and participate in the inhibition of adipocyte differentiation. If this problem can be explained, further study will provide more direct evidence for the interaction between the mesentery and intestine in CD. In addition, the lentivirus was used to regulate the level of pygo2 in *Il‐10* ^*−/−*^ mice, which could not regulate of pygo2 levels in adipocytes organ‐specifically, and explore the effect of pygo2 level in adipocytes on enteritis and mesenteritis in *Il‐10* ^*−/−*^ mice.Our evidence supports a role for Pygo2 as a transcription factor that inhibits adipocyte differentiation and participates in CD mesenteric adipocyte poor differentiation.

## 5 CONCLUSION

Our study found that Pygo2 expression was significantly increased in the MAT of CD patients and *Il‐10* ^*−/−*^ mice. Inhibiting of Pygo2 attenuated the development of MAT lesions in *Il‐10* ^*−/−*^mice by promoting the differentiation of adipocytes, reducing inflammation, and leading to the amelioration of colitis partly through the Axin2/GSK3β pathway. These results enrich the understanding of the crosstalk between MAT and CD colitis.

## AUTHOR CONTRIBUTIONS

Jing Hu designed the research study; Jing Li, Lugen Zuo, Zhijun Geng, Qingqing Li, Yang Cheng and Zi Yang performed the experiments; Jing Li and Lugen Zuo drafted the manuscript; Ruohan Shi, Yueqing Zhou, Wenhu Nie and Yueyue Wang analysed the data; Xiaofeng Zhang, Xue Song and Sitang Ge contributed to the technical support, scientific advice, and manuscript revision. All authors read and approved the final manuscript.

## CONFLICTS OF INTEREST

The authors declare no financial conflicts of interest.

## Supporting information


**Figure S1** Pygo2 intervention effect in MAT and intestinal mucosa. *Il‐10* ^−/−^ mice were infected with lentivirus by intravenous (IV) injection to induce Pygo2 overexpression (OE) or shRNA to induce Pygo2 knockdown (KD); *Il‐10* ^*−/−*^ mice were the model group (8 mice/group). The mice were treated at eight weeks of age for 4 weeks (1 × 10^9^ viral particles/mouse/week). The intervention effect in MAT(A) and intestinal mucosa (B) were verified in *Il‐10* ^*−/−*^ *and Il‐10* ^*−/−*^ Pygo2 OE or Pygo2 KD mice by RT‐qPCR.
**Figure S2** Pygo2 regulates Wnt signalling activation The 3 T3‐L1 cells (8–10 d after differentiation) were infected with lentiviruses to mediate si‐Pygo2 and Pygo2 overexpression (OE‐Pygo2) and stimulated with LPS (100 ng/mL). (A) Western blot analysis of the Wnt target molecular (C‐myc and Cyclin D1). (B) Relative intensities of the proteins. The data are expressed as the mean ± SD. *p < 0.05.

## Data Availability

The data that support the findings of this study are available from the corresponding author upon reasonable request.
