# Supplementary material for: Pygopus2 ameliorates mesenteric adipocyte poor differentiation to alleviate Crohn's disease ‐like colitis via the Axin2/GSK3β pathway
Source: Cell Prolif. 2022 Jun 16;55(10):e13292. doi: 10.1111/cpr.13292 (PMC9528773; doi:10.1111/cpr.13292)
Supplement: Supplementary file 1 — Figure S1 Pygo2 intervention effect in MAT and intestinal mucosa. Il‐10 −/− mice were infected with lentivirus by intravenous (IV) injection to induce Pygo2 overexpression (OE) or shRNA to induce Pygo2 knockdown (KD); Il‐10 −/− mice were the model group (8 mice/group). The mice were treated at eight weeks of age for 4 weeks (1 × 10^9^ viral particles/mouse/week). The intervention effect in MAT(A) and intestinal mucosa (B) were verified in Il‐10 −/− and Il‐10 −/− Pygo2 OE or Pygo2 KD mice by RT‐qPCR. Figure S2 Pygo2 regulates Wnt signalling activation The 3 T3‐L1 cells (8–10 d after differentiation) were infected with lentiviruses to mediate si‐Pygo2 and Pygo2 overexpression (OE‐Pygo2) and stimulated with LPS (100 ng/mL). (A) Western blot analysis of the Wnt target molecular (C‐myc and Cyclin D1). (B) Relative intensities of the proteins. The data are expressed as the mean ± SD. *p < 0.05. [file CPR-55-e13292-s001.docx]

**Supplementary materials**

**Supplementary Figure 1.**


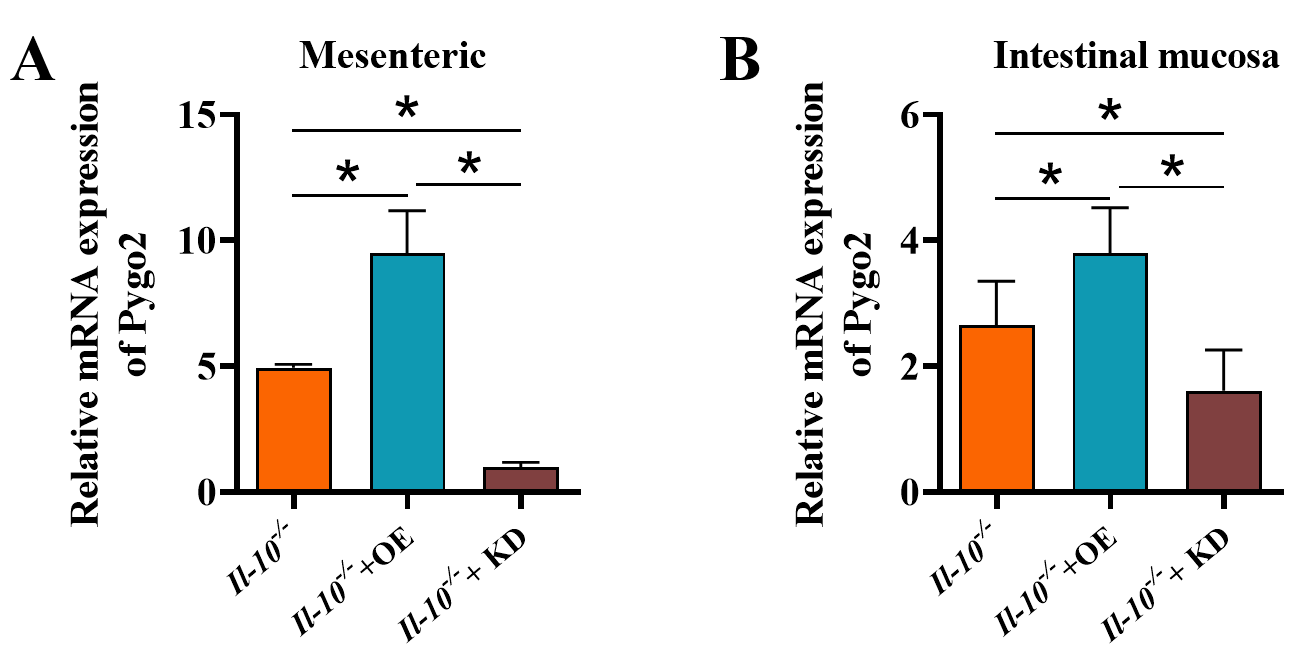


**Supplementary Figure 1. Pygo2 intervention effect in MAT and intestinal mucosa.**

*Il-10*^-/-^ mice were infected with lentivirus by intravenous (IV) injection to induce Pygo2 overexpression (OE) or shRNA to induce Pygo2 knockdown (KD); *Il-10^-/-^* mice were the model group (8 mice/group). The mice were treated at eight weeks of age for 4 weeks (1×10^9^ viral particles/mouse/week). The intervention effect in MAT(A) and intestinal mucosa (B) were verified in *Il-10^-/-^ and Il-10^-/-^* Pygo2 OE or Pygo2 KD mice by RT-qPCR.

**Supplementary Figure 2.**


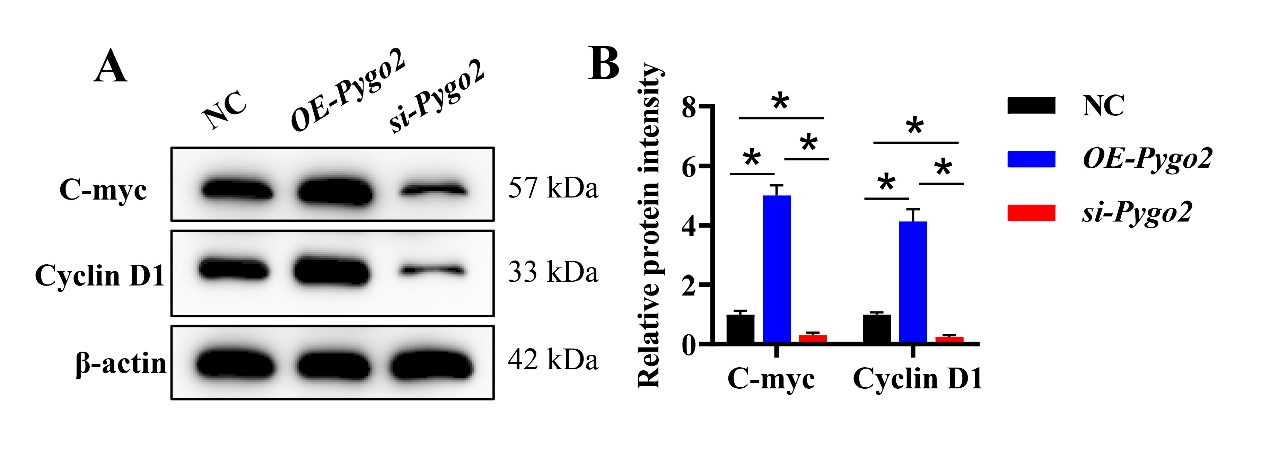


**Supplementary Figure 2. Pygo2 regulates Wnt signaling activation.**

The 3T3-L1 cells (8-10 d after differentiation) were infected with lentiviruses to mediate si-Pygo2 and Pygo2 overexpression (OE-Pygo2) and stimulated with LPS (100 ng/mL). (A) Western blot analysis of the Wnt target molecular (C-myc and Cyclin D1). (B) Relative intensities of the proteins. The data are expressed as the mean ± SD. **P* < 0.05.
